# miRNA and mRNA Integration Network Construction Reveals Novel Key Regulators in Left-Sided and Right-Sided Colon Adenocarcinoma

**DOI:** 10.1155/2019/7149296

**Published:** 2019-04-03

**Authors:** Likun Yang, Lin Li, Junhong Ma, Shimin Yang, Changlin Zou, Xiangyang Yu

**Affiliations:** ^1^Graduate School, Tianjin University of Traditional Chinese Medicine, China; ^2^Department of Gastrointestinal Surgery, Nankai Clinical College of Tianjin Medical University, China; ^3^Department of Gastrointestinal Surgery, Nankai University Nankai Hospital, China

## Abstract

**Background:**

The distinction between right-sided and left-sided colon adenocarcinoma has recently received considerable. This study aims to identify key MicroRNA (miRNA) and mRNAs in right-sided colon adenocarcinoma (RSCOAD) and left-sided colon adenocarcinoma (LSCOAD) by TCGA integration analysis.

**Methods:**

The miRNA and mRNA expression profiles of a large group of patients with RSCOAD and LSCOAD were obtained from TCGA. The differentially expressed miRNAs (DEmiRNAs) and mRNAs (DEmRNAs) were identified by TCGA integration analysis. The optimal diagnostic miRNA biomarkers for RSCOAD and LSCOAD were identified by Boruta algorithm. We established classification models to distinguish RSCOAD and LSCOAD. Protein-protein interaction (PPI) network analysis, DEmiRNA-DEmRNA interaction analysis, and functional annotation were performed. The expression of selected DEmiRNAs and DEmRNAs was validated by qRT-PCR.

**Results:**

A total of 2534 DEmRNAs (940 downregulated and 1594 upregulated mRNAs) and 54 DEmiRNAs (22 downregulated and 32 upregulated miRNAs) between RSCOAD and LSCOAD were identified. The feature selection procedure was to obtain 22 optimal diagnostic miRNAs biomarkers in RSCOAD compared to LSCOAD. The AUC of the random forests model was 0.869 and the specificity and sensitivity of this model were 79% and 84.6%, respectively. Three DEmiRNAs (hsa-miR-224-5p, hsa-miR-155-5p, and hsa-miR-31-5p) and five DEmRNAs (CXCR4, SMAD4, KRAS, FITM2, and PLAGL2) were identified key DEmiRNAs and DEmRNAs in RSCOAD compared to LSCOAD. The qRT-PCR results of CXCR4, FITM2, TFAP2A, ULBP2, hsa-miR-224-5p, and hsa-miR-155-5p were consistent with our integrated analysis.

**Conclusion:**

A total of three DEmiRNAs (hsa-miR-224-5p, hsa-miR-155-5p, and hsa-miR-31-5p) and five DEmRNAs (CXCR4, SMAD4, KRAS, FITM2, and PLAGL2) may be involved in the pathogenesis of RSCOAD and LSCOAD which may make a contribution for understanding mechanisms and developing therapeutic strategies for RSCOAD and LSCOAD.

## 1. Introduction

Colorectal cancer is recognized as one of the most common malignant tumors of cancer-related deaths in worldwide [1]. The human colon has two sides: a right side, containing the ascending and transverse colon, and the left, which is comprised of the descending and sigmoidal colon [2–4]. Many publications pointed out some differences between RSCOAD and LSCOAD regarding epidemiology, clinical presentation, pathology, and genetic mutations [3]. The patients with RSCOAD were older and had more advanced tumor stages, increased tumor sizes, more often poorly differentiated tumors, and different molecular biological tumor patterns. RSCOAD is more prominent in women and LSCOAD is more common in men [5]. Many of studies reported a poorer survival in RSCOAD compared to LSCOAD [6–8]. Hence, it is urgently required to identify accurate indicators in the diagnostic and therapeutic targets in RSCOAD compared to LSCOAD.

MicroRNAs (miRNAs) are a class of small noncoding RNAs with a length of about 18-25nt. miRNAs are recognized as important regulators of gene expression by interacting with the 3′-URT of the target mRNA to inhibit translation or induce degradation [9, 10]. More and more studies have shown that miRNA can be used as an ideal biomarker prognosis for cancer detection and accurate prediction, as well as therapeutic targets [11, 12]. miRNAs regulate the occurrence and development of cancer, including cell proliferation, apoptosis, migration, and invasion [13]. Therefore, identification of RSCOAD and LSCOAD related miRNAs is essential for understanding the occurrence and development of RSCOAD and LSCOAD.

In this study, we used the TCGA integration analysis to study the miRNA and mRNA expression data and uncovered the functional significance of differentially expressed miRNA and mRNA in RSCOAD and LSCOAD.

## 2. Materials and Methods

### 2.1. miRNA and mRNA Gene Expression Profiles in TCGA

The miRNA and mRNA gene expression profiles and clinical data of RSCOAD and LSCOAD were downloaded by the Cancer Genome Atlas (TCGA) (http://tcga-data.nci.nih.gov/). The inclusion criteria for the present study were as follows: (1) Histological Type is colon adenocarcinoma. (2) Anatomic_neoplasm_subdivision Type includes Ascending Colon, Sigmoid Colon, Cecum, and Descending Colon.

### 2.2. Identification of DEmiRNAs and DEmRNAs between RSCOAD and LSCOAD

The undetectable miRNAs and mRNAs (with read count value = 0 in more than 20% RSCOAD case or in more than 20% LSCOAD) were filtered and deleted. The differentially expressed miRNAs (DEmiRNAs) and mRNAs (DEmRNAs) in RSCOAD compared to LSCOAD were performed by R-bioconductor package DESeq2. We used multiple comparisons by using the Benjamini and Hochberg approach to acquire the false discovery rate (FDR). DEmiRNAs and DEmRNAs were defined with the thresholds of FDR < 0.01. Hierarchical clustering analysis of DEmiRNAs and DEmRNAs was further produced by using R package.

### 2.3. Functional Annotation

In order to analyze the function and the potential pathway of DEmiRNAs and target DEmRNAs of DEmiRNAs, the online software GeneCodis was used to conduct the functional annotation, including Gene Ontology (GO) classification and Kyoto Encyclopedia of Genes and Genomes (KEGG) pathway enrichment. FDR<0.05 was defined as the criteria of statistical significance.

### 2.4. Protein-Protein Interaction (PPI) Network Construction

The top100 DEmRNAs were used to build the PPI network by using Biological General Repository for Interaction Datasets (BioGRID) (http://thebiogrid.org/) and Cytoscape (http://www.cytoscape.org/). We used nodes to represent the proteins and edges to represent interactions between two proteins. The nodes and edges indicate proteins and interactions between two proteins, respectively.

### 2.5. Features Selection

Feature selection can readily remove redundant and irrelevant features that contribute to further improving the performance of a classifier. Boruta algorithm was used to minimize errors of random forest model. The optimal feature subset was obtained by using Boruta algorithm (https://cran.r-project.org/web/packages/Boruta/). In the algorithm of Boruta, we used the Z-score as measurement criteria.

### 2.6. DEmiRNA-DEmRNA Interaction Analysis

As miRNAs tend to decrease the expression of their target mRNA, target genes were selected from DEmRNAs expressed inversely with that of miRNA, to subject to further investigation. DEmiRNA-DEmRNA interaction pairs in RSCOAD vs LSCOAD were obtained. Firstly, the correlation between the 22 DEmiRNAs and all of DEmRNAs was analyzed by the pairwise Pearson correlation coefficient. The threshold for DEmiRNA-DEmRNA coexpression pairs was p<0.05 and R<0. Then, the confirmed targeted DEmRNAs of DEmiRNAs were obtained from by miRTarBase. Finally, DEmiRNA-DEmRNA significant negative coexpression pairs overlapped with miRNA-target mRNAs pairs were used to construct the DEmiRNA-DEmRNA coexpression network by using the Cytoscape software (http://www.cytoscape.org/).

### 2.7. Confirmation by qRT-PCR

Fourteen tissues samples of RSCOAD patients (n = 7) and LSCOAD patients (n = 7) were obtained. Informed written consent was obtained from all participants, and research protocols were approved by the ethical committee of our hospital.

Total RNA was extracted with a RNA simple total RNA kit (Tiangen, China). Complementary DNAs were generated using the Fast Quant RT Kit (Tiangen, China). Quantitative real-time PCR were conducted using the Super Real PreMix Plus SYBR Green (Tiangen, China) on ABI 7500 real-time PCR system. Relative quantification of mRNA and miRNA levels was analyzed by using the 2-∆∆Ct method. The PCR primers used are listed in Table 1.

## 3. Results

### 3.1. DEmiRNAs and DEmRNAs between RSCOAD and LSCOAD

**Figure 1:**
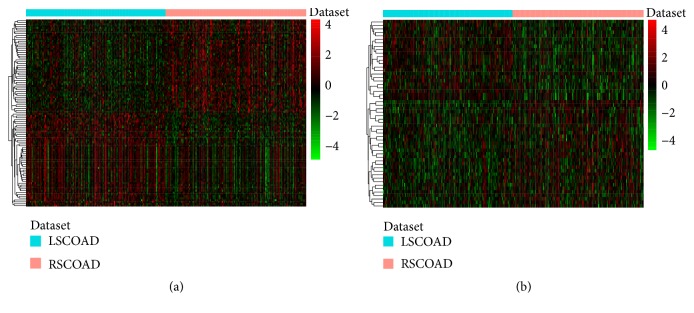
Hierarchical clustering analysis of top 100 DEmRNAs and all of DEmiRNAs between RSCOAD and LSCOAD. (a) DEmRNAs. (b) DEmiRNAs. Row and column represented DEmRNAs/DEmiRNAs and tissue samples, respectively. Orange and light blue color mean the RSCOAD and LSCOAD, respectively. The color scale represented the expression levels. Red color represents that the relative expression level of genes was higher than mean, and green color represents that the relative expression of genes was lower than mean.

We obtained the mRNA and miRNA expression profiles of 151 RSCOAD and 149 LSCOAD patients from TCGA. A total of 2534 DEmRNAs (940 downregulated and 1594 upregulated mRNAs) and 54 DEmiRNAs (22 downregulated and 32 upregulated miRNAs) between RSCOAD and LSCOAD were identified with FDR<0.01. Hierarchical clustering analysis of the top 100 DEmRNAs and all of DEmiRNAs is displayed in Figures 1(a) and 1(b), respectively. The 100 DEmRNAs and all of DEmiRNAs are shown in Supplementary Tables 1 and 2, respectively.

### 3.2. Functional Annotation of DEmRNAs

**Figure 2:**
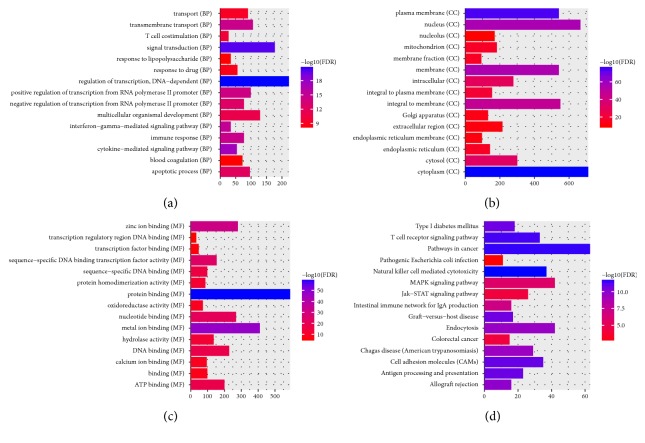
The enrichment GO terms and KEGG pathways of DEmRNAs between RSCOAD and LSCOAD. The x-axis shows -log FDR and y-axis shows GO terms and KEGG pathways. (a) Biological process. (b) Cellular component. (c) Molecular function. (d) KEGG pathways.

All of DEmRNAs were used to perform the GO and KEGG enrichment analysis. According to GO enrichment analysis, regulation of transcription, DNA-dependent (FDR=1.56204E-21), signal transduction (FDR=1.0492E-20), cytoplasm (FDR=1.40992E-78), and protein binding (FDR=8.76318E-60) were significantly enriched GO terms. KEGG pathway enrichment analysis displayed that pathways in cancer (FDR=3.11 E-12), intestinal immune network for IgA production (FDR=1.12E-07), colorectal cancer (FDR=6.51E-05), and pathogenic* Escherichia coli* infection (FDR=2.28E-03) were four significantly enriched pathways. Top 15 most significantly enriched GO and KEGG pathways of DEGs are demonstrated in Figure 2.

### 3.3. PPI Network Construction

**Figure 3:**
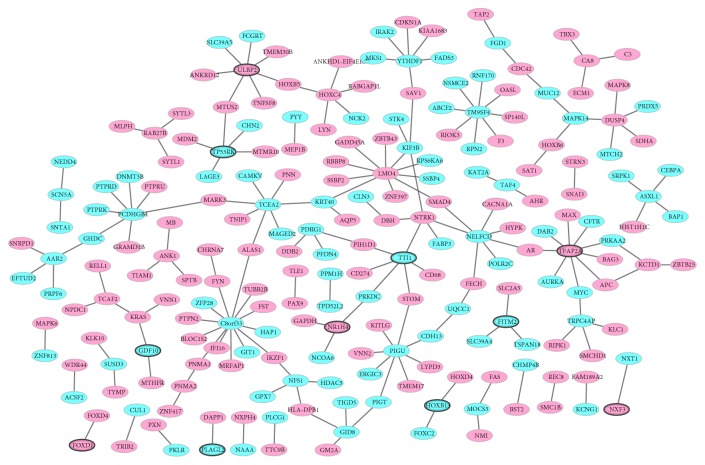
The PPI network construction. Ellipses are used to represent nodes and lines are used to represent edges. Green represents a downward adjustment and red represents a downward adjustment. The solid line means the interaction correlation between proteins. The black border indicates top20Up/Down.

The PPI network of top 100 DEmRNAs consisted of 221 nodes and 194 edges (Figure 3). C8orf33 (degree=13), LMO4 (degree=10), TFAP2A (degree=9), PIGU (degree=8), TM9SF4 (degree=8), and ULBP2 (degree=7) were considered the hub proteins.

### 3.4. Features Selection

**Figure 4:**
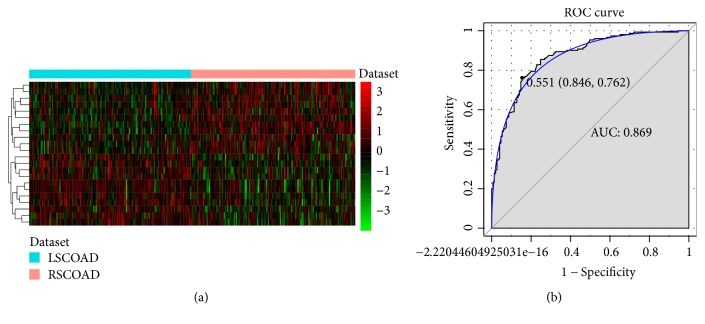
Identification of optimal miRNA biomarkers between RSCOAD and LSCOAD. (a) Hierarchical clustering analysis of 22 DEmiRNAs. Row and column represent DEmiRNAs and tissue samples, respectively. Orange and light blue color mean the RSCOAD and LSCOAD, respectively. The color scale represents the expression levels. Red color represents that the relative expression level of genes was higher than mean, and green color represents that the relative expression of genes was lower than mean. (b) The ROC results of these 22 diagnostic miRNA biomarker based on random forest model.

We obtained 22 DEmiRNAs by algorithms of Boruta (Table 2). Hierarchical clustering analysis of these 22 DEmiRNAs between RSCOAD and LSCOAD is displayed in Figure 4(a). A 10-fold cross-validation result demonstrated that the AUC of the random forests model was 0.869 and the specificity and sensitivity of this model were 79% and 84.6%, respectively (Figure 4(b)).

### 3.5. DEmiRNA-DEmRNA Interaction Network

**Figure 5:**
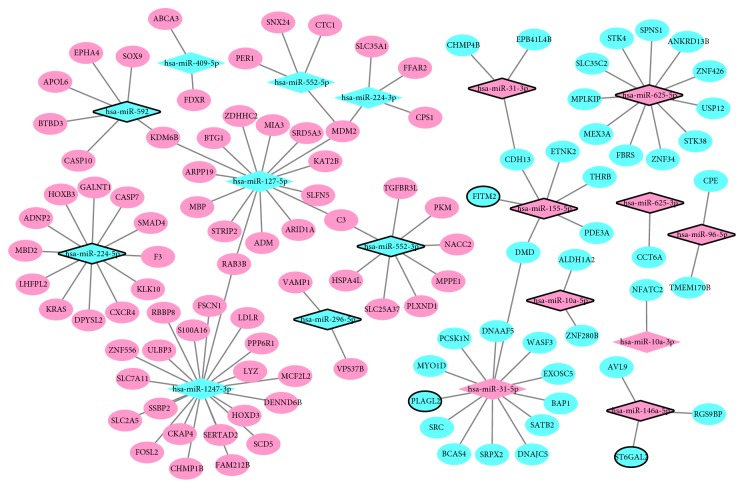
The coexpressed DEmiRNAs-DEmRNAs network. The ellipses and rhombuses represent the DEmRNAs and DEmiRNAs, respectively. Red and blue color represent up- and downregulation, respectively. The solid line means the interaction correlation between proteins. The black border indicates top20 Up and Down, respectively.

miRNAs are negative regulators of their target genes; the expression of targets was negatively associated with miRNAs. According to the miRNA-mRNA expression correlation analysis, we obtained 17563 DEmiRNA-DEmRNA pairs which were negatively correlated (p<0.05, r<0). The 134 upregulated miRNA-mRNA pairs and 124 downregulated miRNA-mRNA pairs were verified by miRTarBase. After overlapping these 258 miRNA-target mRNA pairs and 17564 negative DEmiRNA-DEmRNA coexpression pairs, we obtained 116 DEmiRNA-target DEmRNA pairs including 109 DEmRNAs and 18 DEmiRNAs. Based on the DEmiRNA-target DEmRNA interaction network, hsa-miR-31-5p (degree=13), hsa-miR-224-5p (degree=12), hsa-miR-625-5p (degree=11), and hsa-miR-155-5p (degree=6) were four hub DEmiRNAs (Figure 5).

### 3.6. Functional Annotation of miRNA Targets

**Figure 6:**
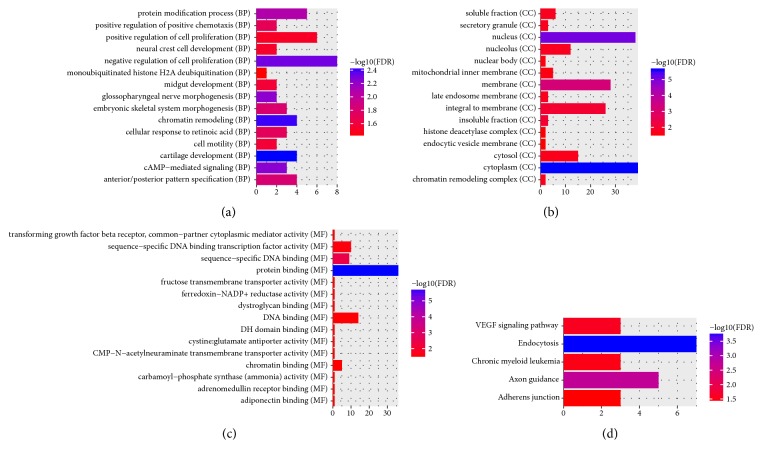
The enrichment GO terms and KEGG pathways of DEmiRNA targets DEmRNAs between RSCOAD and LSCOAD. The x-axis shows -log FDR and y-axis shows GO terms and KEGG pathways. (a) Biological process. (b) Cellular component. (c) Molecular function. (d) KEGG pathways.

After GO enrichment analysis (Figures 6(a)–6(c)), the miRNA targets were significantly enriched in negative regulation of cell proliferation (FDR=0.00490687), cytoplasm (FDR=0.0000024104), and protein binding (FDR=0.00000171927). According to the KEGG pathway enrichment analysis (Figure 6(d)), endocytosis (FDR=0.000168671) and VEGF signaling pathway (FDR=0.0261759) were significantly enriched pathway.

### 3.7. QRT-PCR Confirmation

**Figure 7:**
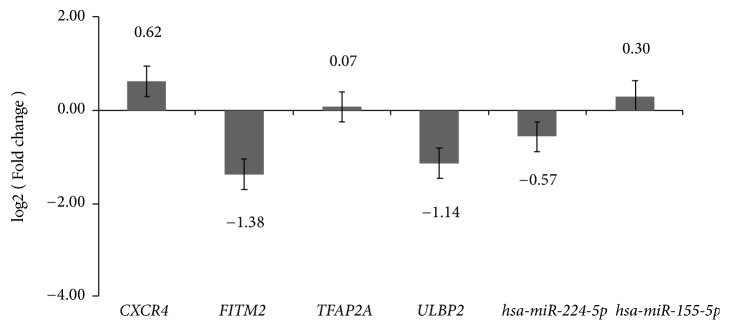
Validation DEmRNAs and DEmiRNAs by qRT-PCR. Fourteen tissues samples of RSCOAD patients (n = 7) and LSCOAD patients (n = 7) were used to perform the validation by qRT-PCR assay. All of the assays were performed three times independently at least. The x-axis shows DEmRNAs or DEmiRNAs and y-axis shows log2(Foldchange). The log2(Foldchange)>0 and log2(Foldchange) <0 indicate upregulation and downregulation, respectively. Statistical significance was assessed by Student's t-test. ^*∗*^P<0.05, ^*∗∗*^P<0.01.

We performed the confirmation of four DEmRNAs (CXCR4, FITM2, TFAP2A, and ULBP2) and two DEmiRNAs (hsa-miR-224-5p and hsa-miR-155-5p) by qRT-PCR. Among them, FITM2, TFAP2A, and ULBP2 were top 10 up/down DEmRNAs, hsa-miR-224-5p and hsa-miR-155-5p top 10 up/down DEmiRNAs. Based on TCGA, FITM2 and hsa-miR-224-5p were downregulated while the other four DEmRNAs or DEmiRNAs (CXCR4, TFAP2A, ULBP2, and hsa-miR-155-5p) were upregulated in RSCOAD to LSCOAD. According to the qRT-PCR results, except for ULBP2, FITM2 and hsa-miR-224-5p were downregulated and CXCR4, TFAP2A, and hsa-miR-155-5p were upregulated which was consistent with the results of TCGA, generally (Figure 7).

## 4. Discussion

The distinction between RSCOAD and LSCOAD has recently received considerable attention [14]. In this study, we performed miRNA and mRNA integrated analysis and obtained 2534 DEGs and 54 DEmiRNAs in RSCOAD patients compared to LSCOAD. A total of 22 DEmiRNAs between RSCOAD and LSCOAD were identified by algorithms of Boruta. According to the functional annotation and DEmiRNA-DEmRNA interaction network, five DEGs (CXCR4, SMAD4, KRAS, FITM2, and PLAGL2) upon the regulation of three DEmiRNAs (hsa-miR-224-5p, hsa-miR-155-5p, and hsa-miR-31-5p) were associated with RSCOAD and LSCOAD.

Hsa-miR-224-5p was downregulated in both TCGA integration analysis and qRT-PCR validation, which was consistent with reports in other cancers of other researchers [15], indicating the TCGA integration analysis results are convincing. According to DEmiRNA-DEmRNA interaction network, hsa-miR-224-5p was coexpressed with CXCR4, SMAD4, and KRAS. C-X-C chemokine receptor type 4 (CXCR4), the receptor for the chemokine stromal cell-derived factor, is one of the members of the chemokine and plays a key role in cancer progression and metastasis [16]. Several reports have found that CXCR4 was upregulated in a variety of cancers, including lung cancer, breast cancer colorectal cancer, and prostate cancer [17–19]. It has been found that the expression levels of CXCR4 correlate with the stage of the tumor, lymph node, and liver metastasis and with a higher expression in the most advanced stages of colorectal cancer [16]. In this study, CXCR4 was upregulated in both TCGA integration analysis and qRT-PCR validation, indicating that the TCGA integration analysis data were reliable. Salovaara et al. have found a strong correlation between the high frequency of SMAD family member 4 (SMAD4) gene mutations and colon cancer distant metastasis [20]. SMAD4 inhibits lymphangiogenesis and migration colon cancer [21]. Recent study has shown that SMAD4 mutation is independently associated with worse outcomes among patients undergoing resection of colorectal liver metastases [22]. KRAS is one of the most common mutated oncogenes in cancer, a powerful promoter of tumorigenesis, a strong induction factor for malignant tumors, and a predictive biomarker of therapeutic response [23]. Hsa-miR-224 was downregulated in the feces from the colorectal cancer patients, which could be an informative biomarker for screening and early diagnosis of colorectal cancer [15]. CXCR4, SMAD4, and KRAS coexpressed with hsa-miR-224-5p were enriched in pathway of Intestinal immune network for IgA production and colorectal cancer. Hence, we inferred that hsa-miR-224-5p-CXCR4/SMAD4/KRAS interactions play a pivotal role in the development of RSCOAD and LSCOAD by regulating pathway of intestinal immune network for IgA production and colorectal cancer.

Hsa-miR-155-5p was upregulated in both TCGA integration analysis and qRT-PCR validation, which was consistent with reports in other cancers of other researchers [24]. MiR-155 directly regulates *β*-catenin at the transcriptional level and promotes the invasion potential of colon cancer cell, which suggests that miR-155 may have a unique potential as a novel biomarker candidate for diagnosis and treatment of tumor metastasis [24]. According to DEmiRNA-DEmRNA interaction network, hsa-miR-155-5p was coexpressed with Fat storage-inducing transmembrane protein 2 (FITM2). FITM2 is a 262-amino acid protein in mammals having six transmembrane domains with both N and C termini facing the cytoplasm. FITM2 causes lethal enteropathy and plays an essential role in regulating intestinal health [24]. In our study, FITM2 was downregulated in both TCGA integration analysis and qRT-PCR validation. Therefore, we hypothesized that hsa-miR-155-5p/FITM2 interactions contributed to distinguishing RSCOAD and LSCOAD.

Hsa-miR-31-5p has been reported as a prognostic biomarker for stage II and III colon cancer [25]. Herein, hsa-miR-31-5p was upregulated in TCGA integration analysis. According to DEmiRNA-DEmRNA interaction network, hsa-miR-31-5p was coexpressed with proto-oncogene, pleomorphic adenoma gene-like 2 (PLAGL2). PLAGL2 is involved in a variety of cancers including colon cancer, acute myeloid leukemia, malignant glioma, and lung adenocarcinoma, and PLAGL2 can function as a tumor suppressor by initiating cell cycle arrest and apoptosis [26]. Hence, we speculated that hsa-miR-31-5p PLAGL2 interactions play a key role in the development of RSCOAD and LSCOAD.

In summary, we identified 2534 DEmRNAs and 54 DEmiRNAs in RSCOAD compared to LSCOAD. The feature selection procedure was to obtain 22 optimal diagnostic miRNAs biomarkers in RSCOAD compared to LSCOAD, among which three DEmiRNAs (hsa-miR-224-5p, hsa-miR-155-5p, and hsa-miR-31-5p) and five DEmRNAs (CXCR4, SMAD4, KRAS, FITM2, and PLAGL2) were identified key DEmiRNAs and DEmRNAs in RSCOAD compared to LSCOAD. However, there are limitations to our study. Firstly, the sample size in the confirmation by qRT-PCR was small and large numbers of samples of RSCOAD and LSCOAD are needed for further research. Secondly, these key DEmRNAs and DEmiRNAs were identified and the function was not studied. Thence, in vivo and in vitro experiments were necessary to illuminate the biological roles of DEmRNAs and DEmiRNAs in the future work.

## Figures and Tables

**Table 1:** Primer sequences used for qRT-PCR.

Name	Sequence (5′to 3′)

GAPDH-F	GGAGCGAGATCCCTCCAAAAT
GAPDH-R	GGCTGTTGTCATACTTCTCATGG
CXCR4-F	ACGCCACCAACAGTCAGAG
CXCR4-R	AGTCGGGAATAGTCAGCAGGA
FITM2-F	CATTCTGACTTTCATCTGGGTGT
FITM2-R	GCTCAGCAAACCAAACAAGGTG
TFAP2A-F	AGGTCAATCTCCCTACACGAG
TFAP2A-R	GGAGTAAGGATCTTGCGACTGG
ULBP2-F	GTGGTGGACATACTTACAGAGC
ULBP2-R	CTGCCCATCGAAACTGAACTG
hsa-miR-224-5p	TCAAGTCACTAGTGGTTCCGTTTAG
hsa-miR-155-5p	TTAATGCTAATCGTGATAGGGGTT

**Table 2:** 22 DEmiRNAs screened by Boruta.

Symbol	log2FoldChange	P.value	FDR	Up_Down

hsa-miR-10b-5p	9.17E-01	1.78E-24	8.62E-22	Up
hsa-miR-10b-3p	9.87E-01	2.07E-19	5.00E-17	Up
hsa-miR-155-5p	7.42E-01	2.25E-13	3.62E-11	Up
hsa-miR-146a-5p	7.06E-01	1.04E-10	1.26E-08	Up
hsa-miR-625-5p	6.84E-01	1.26E-09	1.22E-07	Up
hsa-miR-296-5p	-9.27E-01	1.75E-08	1.41E-06	Down
hsa-miR-592	-8.27E-01	8.22E-08	5.67E-06	Down
hsa-miR-625-3p	5.79E-01	9.55E-08	5.76E-06	Up
hsa-miR-96-5p	4.82E-01	1.22E-07	5.89E-06	Up
hsa-miR-10a-5p	5.21E-01	8.40E-07	3.38E-05	Up
hsa-miR-31-3p	9.87E-01	7.81E-07	3.38E-05	Up
hsa-miR-31-5p	9.38E-01	1.95E-06	6.73E-05	Up
hsa-miR-10a-3p	5.03E-01	7.51E-06	2.27E-04	Up
hsa-miR-224-5p	-4.51E-01	2.25E-05	5.17E-04	Down
hsa-miR-552-3p	-5.51E-01	5.35E-05	9.72E-04	Down
hsa-miR-1247-5p	-6.40E-01	5.83E-05	9.72E-04	Down
hsa-miR-1247-3p	-6.46E-01	7.13E-05	1.15E-03	Down
hsa-miR-224-3p	-4.52E-01	7.50E-05	1.17E-03	Down
hsa-miR-409-5p	-3.66E-01	4.16E-04	4.46E-03	Down
hsa-miR-452-5p	-3.40E-01	4.34E-04	4.46E-03	Down
hsa-miR-127-5p	-3.30E-01	4.31E-04	4.46E-03	Down
hsa-miR-552-5p	-4.53E-01	6.62E-04	6.37E-03	Down

## Data Availability

The data used to support the findings of this study are included within the article.

## Supplementary Materials

Supplementary Materials The Supplementary Table of top 100 DEmRNAs and all of DEmiRNAs. Supplementary Table 1 shows top of 100 DEmRNAs, and Supplementary Table 2 shows all of DEmiRNAs.
