# Supplementary material for: miRNA and mRNA Integration Network Construction Reveals Novel Key Regulators in Left-Sided and Right-Sided Colon Adenocarcinoma
Source: Biomed Res Int. 2019 Apr 3;2019:7149296. doi: 10.1155/2019/7149296 (PMC6470432; doi:10.1155/2019/7149296)
Supplement: Supplementary Materials — The Supplementary Table of top 100 DEmRNAs and all of DEmiRNAs. Supplementary Table 1 shows top of 100 DEmRNAs, and Supplementary Table 2 shows all of DEmiRNAs. [file 7149296.f1.docx]

**Supplementary Table 1 Top of 100 DEmRNAs**

| **mRNA** | **log2FoldChange** | **pvalue** | **FDR** | **Up/down** |
| --- | --- | --- | --- | --- |
| GDF10 | -2.399362016 | 1.88E-35 | 2.96E-31 | Down |
| HOXB13 | -1.511121861 | 5.16E-29 | 4.06E-25 | Down |
| NXF3 | 2.195478728 | 1.99E-28 | 1.05E-24 | Up |
| ULBP2 | 1.507067119 | 2.42E-25 | 9.54E-22 | Up |
| HOXB2 | 1.192627517 | 4.04E-23 | 1.27E-19 | Up |
| ERFE | 1.417589816 | 7.48E-23 | 1.96E-19 | Up |
| FITM2 | -0.835602785 | 8.49E-22 | 1.91E-18 | Down |
| MUC5AC | 1.840243401 | 2.69E-21 | 5.30E-18 | Up |
| FOXD1 | 1.745970523 | 6.82E-21 | 1.19E-17 | Up |
| TFAP2A | 1.374457732 | 8.49E-21 | 1.34E-17 | Up |
| NR1H4 | 1.646980612 | 2.79E-19 | 4.00E-16 | Up |
| POFUT1 | -0.720900031 | 4.30E-19 | 5.64E-16 | Down |
| EMX1 | 1.719903399 | 6.83E-19 | 8.28E-16 | Up |
| WNT7B | 1.5287483 | 8.54E-19 | 9.61E-16 | Up |
| DUSP4 | 1.333647925 | 8.03E-18 | 8.43E-15 | Up |
| ST6GAL2 | -1.492510923 | 1.56E-17 | 1.54E-14 | Down |
| TTI1 | -0.493389481 | 2.92E-17 | 2.71E-14 | Down |
| RAB27B | 1.183281939 | 4.30E-17 | 3.77E-14 | Up |
| TTC9B | 1.087534302 | 5.13E-17 | 4.26E-14 | Up |
| GABRP | 1.536456186 | 5.75E-17 | 4.53E-14 | Up |
| PLAGL2 | -0.706985568 | 1.77E-16 | 1.25E-13 | Down |
| TRIM7 | 1.367229967 | 1.78E-16 | 1.25E-13 | Up |
| ZNF880 | -1.096945489 | 1.90E-16 | 1.25E-13 | Down |
| TRIB2 | 0.905721785 | 1.91E-16 | 1.25E-13 | Up |
| PXMP4 | -0.555234126 | 3.90E-16 | 2.46E-13 | Down |
| ADGRF1 | 1.401985946 | 4.39E-16 | 2.66E-13 | Up |
| ANK1 | 1.071044197 | 4.74E-16 | 2.77E-13 | Up |
| HOXC4 | 1.351530708 | 4.92E-16 | 2.77E-13 | Up |
| CXCL17 | 1.596221777 | 9.66E-16 | 5.25E-13 | Up |
| TP53RK | -0.616755629 | 1.03E-15 | 5.42E-13 | Down |
| FABP3 | -1.29277047 | 1.07E-15 | 5.44E-13 | Down |
| ADGRG6 | 1.042684499 | 1.27E-15 | 6.25E-13 | Up |
| MYRFL | 1.44766634 | 1.51E-15 | 7.21E-13 | Up |
| VNN1 | 1.39675077 | 1.81E-15 | 8.40E-13 | Up |
| TM4SF4 | 1.452721462 | 1.96E-15 | 8.82E-13 | Up |
| SNAI3 | 0.879328773 | 2.45E-15 | 1.07E-12 | Up |
| PYY | -1.527655209 | 2.57E-15 | 1.09E-12 | Down |
| FGD1 | -0.762671863 | 2.65E-15 | 1.10E-12 | Down |
| CAMK2N2 | 1.28452488 | 2.75E-15 | 1.11E-12 | Up |
| PAX9 | 1.181938196 | 2.89E-15 | 1.14E-12 | Up |
| HOXB6 | 0.956754781 | 3.18E-15 | 1.22E-12 | Up |
| SMC1B | 1.2876588 | 3.30E-15 | 1.24E-12 | Up |
| ZNF813 | -1.150626878 | 3.59E-15 | 1.30E-12 | Down |
| PCDHGB4 | -1.348198362 | 3.62E-15 | 1.30E-12 | Down |
| HOXD3 | 1.094160707 | 4.22E-15 | 1.48E-12 | Up |
| MEP1B | 1.355913646 | 4.52E-15 | 1.55E-12 | Up |
| YTHDF1 | -0.383871984 | 5.11E-15 | 1.71E-12 | Down |
| BST2 | 1.091999164 | 5.28E-15 | 1.73E-12 | Up |
| SUSD3 | -1.017091336 | 9.17E-15 | 2.95E-12 | Down |
| KCTD1 | 0.868876774 | 1.19E-14 | 3.73E-12 | Up |
| TPD52L2 | -0.42802416 | 1.31E-14 | 4.03E-12 | Down |
| SYNGR3 | 1.159381197 | 1.42E-14 | 4.30E-12 | Up |
| TM9SF4 | -0.462817743 | 1.61E-14 | 4.79E-12 | Down |
| ONECUT2 | 1.099950013 | 2.49E-14 | 7.25E-12 | Up |
| NXPH4 | 1.426618471 | 2.77E-14 | 7.95E-12 | Up |
| TCN1 | 1.462350666 | 2.93E-14 | 8.23E-12 | Up |
| AQP5 | 1.522462595 | 3.11E-14 | 8.58E-12 | Up |
| LAG3 | 1.017554639 | 3.31E-14 | 8.98E-12 | Up |
| CTSE | 1.45657101 | 3.60E-14 | 9.60E-12 | Up |
| PKLR | -1.343600522 | 5.31E-14 | 1.39E-11 | Down |
| DBH | 1.144322675 | 5.49E-14 | 1.42E-11 | Up |
| TRPC4AP | -0.394538002 | 5.89E-14 | 1.50E-11 | Down |
| C8orf33 | -0.661676128 | 5.99E-14 | 1.50E-11 | Down |
| SYS1 | -0.40500268 | 6.44E-14 | 1.59E-11 | Down |
| UQCC1 | -0.431733285 | 7.47E-14 | 1.81E-11 | Down |
| MUC6 | 1.449984148 | 7.96E-14 | 1.90E-11 | Up |
| SLC22A31 | -1.394491932 | 8.72E-14 | 2.05E-11 | Down |
| CHRNA7 | 1.320372809 | 9.06E-14 | 2.10E-11 | Up |
| AAR2 | -0.417682347 | 9.59E-14 | 2.19E-11 | Down |
| TAF4 | -0.470072114 | 1.34E-13 | 3.01E-11 | Down |
| CHN2 | -0.836213607 | 1.40E-13 | 3.08E-11 | Down |
| SCN5A | -1.18221502 | 1.41E-13 | 3.08E-11 | Down |
| LMO4 | 0.702655371 | 1.53E-13 | 3.30E-11 | Up |
| PNMA2 | 1.136919124 | 1.60E-13 | 3.41E-11 | Up |
| CLDN18 | 1.49709616 | 1.92E-13 | 4.04E-11 | Up |
| CX3CL1 | 0.896010135 | 2.45E-13 | 5.07E-11 | Up |
| TCAF2 | 0.787485576 | 3.21E-13 | 6.51E-11 | Up |
| CA8 | 1.237882969 | 3.23E-13 | 6.51E-11 | Up |
| KIF3B | -0.421761068 | 3.27E-13 | 6.51E-11 | Down |
| ZNF853 | -0.899496791 | 3.40E-13 | 6.70E-11 | Down |
| HMSD | 1.218496799 | 3.53E-13 | 6.87E-11 | Up |
| NFS1 | -0.414277313 | 3.73E-13 | 7.17E-11 | Down |
| SLITRK6 | 1.389943494 | 4.21E-13 | 7.98E-11 | Up |
| FOXD4 | 0.997512338 | 5.16E-13 | 9.67E-11 | Up |
| AMT | -0.864647538 | 5.39E-13 | 9.98E-11 | Down |
| ASXL1 | -0.436238565 | 5.70E-13 | 1.04E-10 | Down |
| SLC35C2 | -0.401747356 | 5.99E-13 | 1.08E-10 | Down |
| OSER1 | -0.562085525 | 6.88E-13 | 1.23E-10 | Down |
| NELFCD | -0.482180786 | 6.99E-13 | 1.24E-10 | Down |
| PIGU | -0.486236644 | 7.17E-13 | 1.24E-10 | Down |
| KCNG1 | -1.182257319 | 7.17E-13 | 1.24E-10 | Down |
| FSIP1 | -1.072628237 | 7.78E-13 | 1.31E-10 | Down |
| SP140 | 0.94426955 | 7.79E-13 | 1.31E-10 | Up |
| UGDH | 0.509022428 | 7.82E-13 | 1.31E-10 | Up |
| TCEA2 | -0.813136756 | 8.04E-13 | 1.33E-10 | Down |
| DUSP15 | -1.036206903 | 8.58E-13 | 1.41E-10 | Down |
| GID8 | -0.378804701 | 8.71E-13 | 1.41E-10 | Down |
| PDE10A | 1.012221962 | 8.91E-13 | 1.43E-10 | Up |
| FAM46A | 0.659761821 | 9.15E-13 | 1.45E-10 | Up |
| SYN3 | -1.239079689 | 9.19E-13 | 1.45E-10 | Down |

**Supplementary Table 2 All of DEmiRNAs**

| **miRNA** | **log2FoldChange** | **pvalue** | **FDR** | **Up/down** |
| --- | --- | --- | --- | --- |
| hsa-miR-10b-5p | 0.917258566 | 1.78E-24 | 8.62E-22 | Up |
| hsa-miR-10b-3p | 0.987351002 | 2.07E-19 | 5.00E-17 | Up |
| hsa-miR-155-5p | 0.74212202 | 2.25E-13 | 3.62E-11 | Up |
| hsa-miR-146a-5p | 0.706062272 | 1.04E-10 | 1.26E-08 | Up |
| hsa-miR-625-5p | 0.68407717 | 1.26E-09 | 1.22E-07 | Up |
| hsa-miR-296-5p | -0.926592207 | 1.75E-08 | 1.41E-06 | Down |
| hsa-miR-592 | -0.826960837 | 8.22E-08 | 5.67E-06 | Down |
| hsa-miR-625-3p | 0.579154705 | 9.55E-08 | 5.76E-06 | Up |
| hsa-miR-582-5p | 0.465427933 | 1.19E-07 | 5.89E-06 | Up |
| hsa-miR-96-5p | 0.481530337 | 1.22E-07 | 5.89E-06 | Up |
| hsa-miR-31-3p | 0.987301877 | 7.81E-07 | 3.38E-05 | Up |
| hsa-miR-10a-5p | 0.521227483 | 8.40E-07 | 3.38E-05 | Up |
| hsa-miR-330-5p | 0.515108923 | 1.06E-06 | 3.95E-05 | Up |
| hsa-miR-31-5p | 0.938334425 | 1.95E-06 | 6.73E-05 | Up |
| hsa-miR-92b-3p | 0.474468449 | 2.16E-06 | 6.97E-05 | Up |
| hsa-miR-10a-3p | 0.502683612 | 7.51E-06 | 0.000226826 | Up |
| hsa-miR-582-3p | 0.417370887 | 1.00E-05 | 0.000284867 | Up |
| hsa-miR-20a-3p | -0.533033085 | 1.39E-05 | 0.000371923 | Down |
| hsa-miR-29c-3p | 0.454793277 | 2.15E-05 | 0.000517332 | Up |
| hsa-miR-342-3p | 0.417756417 | 2.20E-05 | 0.000517332 | Up |
| hsa-miR-224-5p | -0.450886138 | 2.25E-05 | 0.000517332 | Down |
| hsa-miR-19a-3p | -0.520824115 | 2.67E-05 | 0.000586912 | Down |
| hsa-miR-6761-5p | -0.524975195 | 2.84E-05 | 0.000597307 | Down |
| hsa-miR-362-5p | -0.505753843 | 4.11E-05 | 0.000826602 | Down |
| hsa-miR-188-5p | -0.485946304 | 4.94E-05 | 0.000955204 | Down |
| hsa-miR-146b-3p | 0.408954354 | 5.44E-05 | 0.000971728 | Up |
| hsa-miR-552-3p | -0.550974771 | 5.35E-05 | 0.000971728 | Down |
| hsa-miR-503-5p | -0.568814795 | 5.82E-05 | 0.000971728 | Down |
| hsa-miR-1247-5p | -0.640440001 | 5.83E-05 | 0.000971728 | Down |
| hsa-miR-1247-3p | -0.646468481 | 7.13E-05 | 0.001147856 | Down |
| hsa-miR-224-3p | -0.451719427 | 7.50E-05 | 0.00116847 | Down |
| hsa-miR-452-3p | -0.432805687 | 8.46E-05 | 0.001276629 | Down |
| hsa-miR-150-5p | 0.475967729 | 8.85E-05 | 0.001295855 | Up |
| hsa-miR-196b-5p | -0.530777582 | 9.18E-05 | 0.001304385 | Down |
| hsa-miR-5586-5p | 0.542539976 | 9.66E-05 | 0.001332825 | Up |
| hsa-miR-142-5p | 0.408590793 | 0.00012629 | 0.001694386 | Up |
| hsa-miR-19b-3p | -0.367046789 | 0.000145822 | 0.001903564 | Down |
| hsa-miR-33a-5p | -0.516987821 | 0.000155346 | 0.001974527 | Down |
| hsa-miR-183-3p | 0.545057719 | 0.000200112 | 0.002433242 | Up |
| hsa-miR-146b-5p | 0.336503984 | 0.000201511 | 0.002433242 | Up |
| hsa-miR-140-5p | 0.220338117 | 0.000245093 | 0.002887313 | Up |
| hsa-miR-375 | 0.451793793 | 0.000261381 | 0.002964745 | Up |
| hsa-miR-215-5p | 0.547562976 | 0.000263942 | 0.002964745 | Up |
| hsa-miR-182-5p | 0.356542567 | 0.000409806 | 0.004461981 | Up |
| hsa-miR-409-5p | -0.36566714 | 0.000415713 | 0.004461981 | Down |
| hsa-miR-127-5p | -0.330423266 | 0.000430702 | 0.00446467 | Down |
| hsa-miR-452-5p | -0.339921249 | 0.00043445 | 0.00446467 | Down |
| hsa-miR-132-3p | 0.318009772 | 0.000484115 | 0.004871404 | Up |
| hsa-miR-215-3p | 0.674874502 | 0.000562546 | 0.005545092 | Up |
| hsa-miR-4772-3p | 0.401697275 | 0.000672429 | 0.006368301 | Up |
| hsa-miR-552-5p | -0.452518472 | 0.000662209 | 0.006368301 | Down |
| hsa-miR-183-5p | 0.346798831 | 0.000700102 | 0.006502867 | Up |
| hsa-miR-3614-5p | 0.47941969 | 0.000724722 | 0.006604543 | Up |
| hsa-miR-3131 | -0.547004942 | 0.000744662 | 0.006660586 | Down |
